# Association between out-of-hours discharge and mortality in adult patients leaving critical care

**DOI:** 10.1186/cc14644

**Published:** 2015-03-16

**Authors:** S Edie, K Burt, J Paddle

**Affiliations:** 1 Royal Cornwall Hospital, Truro, UK

## Introduction

Out-of-hours (OOH) discharge from critical care is associated with a significantly increased mortality rate in Australasia [1]. In the UK, daytime discharges from critical care are considered a core standard [2]. We sought to assess the impact of OOH discharge from critical care on mortality in a large general ICU, where operational pressures appear to have led to a high rate of OOH discharges.

## Methods

Retrospective data for all patients admitted to our ICU from April 2007 to September 2014 were recorded, using routinely collected data from our databases. Adult patients (>15 years) discharged from their first ICU admission during each hospital stay (episode) were included. Patients that died on the unit and those discharged for palliative care were excluded. Patients transferred to other centres were no longer subject to discharge within our control and were therefore also excluded. Patients discharged directly home from ICU were excluded. We defined OOH discharges as those occurring between 22:00 and 06:59, a standard definition in UK practice. Mortality status at the time of hospital discharge for each episode was used. We also recorded the readmission rate to ICU. The relative risk (RR) for OOH mortality and readmission was calculated. Statistical significance was accepted at *P *< 0.05.

## Results

Of 4,476 index cases, 714 died on the unit and 80 were discharged for palliative care. A total of 490 patients were excluded for transfer to other centres and discharge directly home. Data were missing for three patients, which left 3,189 records for analysis. In total, 2,711 patients were discharged during daytime hours, of which 145 (5.35%) died. A total of 478 patients were discharged at night, 40 died (8.37%). The RR for OOH mortality was 1.56 (95% CI = 1.12 to 2.19, *P *= 0.0091). Readmission rate was 5.2% by day, 6.1% at night. The RR for readmission was 1.17 (95% CI = 0.79 to 1.72, *P *= 0.436).

## Conclusion

Our data demonstrate an association between critical care discharge time and mortality, to a statistically significant level. Due to the retrospective observational nature of the study, causation cannot be assumed; however, a number of factors may contribute to the increased risk of harm to patients discharged from the ICU at night. Further work will focus on annual OOH mortality trends, thereby gaining an insight into whether bed occupancy demands impact on the necessity for nighttime discharges.
